# Epidemic characterization and molecular genotyping of *Shigella flexneri* isolated from calves with diarrhea in Northwest China

**DOI:** 10.1186/s13756-017-0252-6

**Published:** 2017-09-06

**Authors:** Zhen Zhu, Mingze Cao, Xuzheng Zhou, Bing Li, Jiyu Zhang

**Affiliations:** Key Laboratory of New Animal Drug Project of Gansu Province, Key Laboratory of Veterinary Pharmaceutical Development of Ministry of Agriculture, Lanzhou Institute of Husbandry and Pharmaceutical Sciences of CAAS, Jiangouyan, Qilihe District, Lanzhou, People’s Republic of China

**Keywords:** *Shigella flexneri*, Antimicrobial susceptibility, Resistant

## Abstract

**Background:**

The widespread presence of antibiotics resistance genes in pathogenic bacteria can cause enormous problems. Food animals are one of the main reservoirs of intestinal pathogens that pose a potential risk to human. Analyzing the epidemiological characteristics and resistance patterns of *Shigella flexneri* in calves is necessary for animal and human health.

**Methods and results:**

A total of 54 *Shigella flexneri* isolates, including six serotypes (1a, 2a, 2b, 4a, 6 and Xv), were collected from 837 fecal samples obtained from 2014 to 2016. We performed pulsed-field gel electrophoresis (PFGE) and applied the restriction enzyme *Not*I to analyze the genetic relatedness among the 54 isolates and to categorize them into 31 reproducible and unique PFGE patterns. According to the results of antimicrobial susceptibility tests, all 26 *Shigella flexneri* 2a serotypes were resistant to cephalosporin and/or fluoroquinolones. The genes *bla*_*TEM*-*1*_, *bla*_*OXA*-*1*_, and *bla*_*CTX*-*M*-*14*_ were detected in 19 cephalosporin-resistant *S. flexneri* 2a isolates. Among 14 fluoroquinolone-resistant isolates, the *aac(6*′*)-Ib-cr* gene was largely present in each strain, followed by *qnrS* (5). Only one ciprofloxacin-resistant isolate harbored the *qepA* gene. Sequencing the quinolone resistance determining regions (QRDRs) of the fluoroquinolone-resistant isolates revealed two point mutations in *gyrA* (S83 L, D87N/Y) and a single point mutation in *parC* (S80I). Interestingly, two *gyrA* (D87N/Y) strains were resistant to ciprofloxacin.

**Conclusions:**

The current study enhances our knowledge of *Shigella* in cattle, although continual surveillance is necessary for the control of shigellosis. The high level of cephalosporin and/or fluoroquinolone resistance in *Shigella* warns us of a potential risk to human and animal health.

## Background

The majority of Enterobactericeae family bacteria, including *Salmonella*, *E. coli* and *Shigella* spp., the major etiological agent of diarrheal disease, are a global public health burden, particularly in low-income countries [1–3]. *Shigella* is phylogenetically distinct from several independent *E. coli* strains and has evolved through convergent evolution [4]. The genus *Shigella* consists of four subgroups differentiated according to their biochemical and serological properties: A (*S. dysenteriae*), B (*S. flexneri*), C (*S. boydii*), and D (*S. sonnei*). All four species of *Shigella* cause shigellosis, but *S. flexneri* is the predominant subgroup found in developing countries, whereas *S. sonnei* is found in industrialized countries [5]. The first *Shigella* species identified was *S. dysenteriae*, followed by *S. flexneri* at the end of the 19th century. Shigellosis became a notorious and widespread epidemic during World War 1 with the transmission of *S. flexneri* strain NCTC1, a 2a lineage [6, 7]. Based on the differing structural characteristics of the antigenic determinants of the O antigen, *S. flexneri* is divided into no fewer than 20 serotypes: 1a, 1b, 1c, 1d, 2a, 2b, 2v, 3a, 3b, 4a, 4av, 4b, 4c, 5a, 5b, X, Xv, Y, Yv, 6, and 7b [8, 9].

Given that shigellosis is a global public health burden, previous studies have focused on the human gastrointestinal pathogens but have ignored animal groups. *Shigella* spp. infect and also cause corresponding clinical symptoms in monkeys, cows, pigs, chickens and other animals [10–13]. Indeed, animals that live in environments characterized by poor sanitary hygiene, restricted access to clean drinking water and long-term exposure to contaminated food are prone to dysentery [14, 15]. Many antibiotics are used to control disease and promote growth during the breeding process, leading to the widespread dissemination of antibiotic resistance genes (ARGs). The spread of drug resistance among pathogenic bacteria in humans and animals may be disastrous.

The present study investigated the *Shigella* epidemic in cows in the northwest region of China. *S. flexneri* 2a was first isolated from a yak with diarrhea in Tibet in 2014. In this study, we used pulsed-field gel electrophoresis (PFGE) to analyze the relationships among *S. flexneri* isolates and tested for antimicrobial susceptibility patterns. Our results will help prevent diarrhea in calves and will assist in the selection of effective antibiotics against *Shigella*.

## Methods

### Bacterial isolation and identification

Fresh stool samples were isolated from 2014 to 2016 in Northwest China (Gansun, Shanxi, Qinghai, Xinjiang and Tibet) from calves (3 to 20 days) with diarrhea. Samples were stored in transport medium, cultured directly on *Salmonella-Shigella* (SS) agar and incubated at 37 °C for 24 h to select for *Shigella*. Resultant colonies (colorless, semitransparent, smooth, and moist circular) [16] were picked and grown at 37 °C for 24 h on MacConkey (MAC) Agar to verify identity. Colonies were selected and cultured in brain heart infusion broth at 37 °C for 5 h with shaking at 250 rpm. All isolates were confirmed using API20E test strips (bioMerieux Vitek, Marcy-l’ Etoile, France) according to the manufacturer’s recommendations. *Shigella* was serotyped using a commercially available kit (Denka Seiken, Tokyo, Japan) and confirmed by PCR [17].

### Antimicrobial susceptibility testing

The antimicrobial susceptibility of *S. flexneri* isolates was determined via the Kirby–Bauer disc-diffusion method in accordance with the guidelines of the Clinical and Laboratory Standards Institute (CLSI) [18].

The antibiotic discs (OXOID, UK) included penicillin G (P, 10 μg), ampicillin (AMP, 10 μg), amoxicillin/clavulanic acid (AMC, 30 μg), cephalothin (KF, 30 μg), cephazolin (KZ, 30 μg), cefamandole (MA, 30 μg), cefoxitin (FOX, 30 μg), ceftriaxone (CRO, 30 μg), cefotaxime (CTX, 30 μg), cefoperazone (CFP, 75 μg), cefepime (FEP, 30 μg), meropenem (MEM, 10 μg), imipenem (IPM, 10 μg), norfloxacin (NOR, 10 μg), enrofloxacin (ENR, 5 μg), levofloxacin (LEV, 5 μg), ciprofloxacin (CIP, 5 μg), erythromycin (E, 15 μg), chloramphenicol (C, 30 μg), tetracycline (TE, 30 μg), streptomycin (S, 10 μg), gentamicin (CN, 10 μg), and amikacin (AK, 30 μg). *E. coli* strain ATCC25922 was used as a quality control strain in each test batch.

### PCR amplification of ARGs

We performed PCR assays that targeted 24 different ARGs using the primers described in Table 1. To determine the underlying resistance mechanism of β-lactam antibiotics, we amplified extended-spectrum β-lactamase (ESBL) genes, specifically *bla*_*CTX-M*_, *bla*_*SHV*_, *bla*_*TEM*_, and *bla*_*OXA*_, as well as *ampC* genes, specifically *bla*_*MOX*_, *bla*_*FOX*_, *bla*_*DHA*_, *bla*_*CIT*_, *bla*_*ACC*_, and *bla*_*MIR*_ [19–21]. Plasmid-mediated quinolone resistance (PMQR) determinant genes, including *qnrA*, *qnrB*, *qnrD*, *qnrS*, *qepA* and *aac(6′)-Ib-cr* and four quinolone resistance determining region (QRDR) genes as well as DNA gyrase (*gyrA*,*gyrB*) and topoisomerase IV (*parC*,*parE*) were amplified to determine the underlying mechanism of quinolone resistance [16, 21–23]. The PCR fragments were sequenced after purification and compared to sequences in GenBank.

**Table 1:** Primers for the detection of antibiotic resistance genes

Target	Primer sequence (5′ to 3′)	Amplicon size (bp)	Reference
β-lactamase			
*bla*_*CTX*-*M*-*1*_	F: GGTTAAAAAATCACTGCGTC	873	Cui et al., 2015 [16]
	R: TTACAAACCGTCGGTGACGA		
*bla*_*CTX*-*M*-*2*_	F: CGACGCTACCCCTGCTATT	552	Zong et al., 2008 [17]
	R: CCAGCGTCAGATTTTTCAGG		
*bla*_*CTX*-*M*-*8*_	F: TCGCGTTAAGCGGATGATGC	689	Zong et al., 2008 [17]
	R: AACCCACGATGTGGGTAGC		
*bla*_*CTX*-*M*-*9*_	F: AGAGTGCAACGGATGATG	868	Cui et al., 2015 [16]
	R: CCAGTTACAGCCCTTCGG		
*bla*_*CTX*-*M*-*25*_	F: TTGTTGAGTCAGCGGGTTGA	497	Liu et al., 2015 [18]
	R: GCGCGACCTTCCGGCCAAAT		
*bla*_*SHV*_	F: CGCCGGGTTATTCTTATTTGTCGC	1015	Zong et al., 2008 [17]
	R: TCTTTCCGATGCCGCCGCCAGTCA		
*bla*_*TEM*_	F: ATGAGTATTCAACTTTCCG	876	This study
	R: CCAATGCTTAATCAGTGAG		
*bla*_*OXA*_	F: ATTAAGCCCTTTACCAAACCA	890	Cui et al., 2015 [16]
	R: AAGGGTTGGGCGATTTTGCCA		
*bla*_*MOX*_	F: GCTGCTCAAGGAGCACAGGAT	520	Cui et al., 2015 [16]
	R: CACATTGACATAGGTGTGGTGC		
*bla*_*FOX*_	F: AACATGGGGTATCAGGGAGATG	190	Cui et al., 2015 [16]
	R: CAAAGCGCGTAACCGGATTGG		
*bla*_*DHA*_	F: AACTTTCACAGGTGTGCTGGGT	405	Cui et al., 2015 [16]
	R: CCGTACGCATACTGGCTTTGC		
*bla*_*CIT*_	F: TGGCCAGAACTGACAGGCAAA	462	Cui et al., 2015 [16]
	R: TTTCTCCTGAACGTGGCTGGC		
*bla*_*ACC*_	F: AACAGCCTCAGCAGCCGGTTA	346	Cui et al., 2015 [16]
	R: TTCGCCGCAATCATCCCTAGC		
*bla*_*MIR*_	F: TCGGTAAAGCCGATGTTGCGG	302	Cui et al., 2015 [16]
	R: CTTCCACTGCGGCTGCCAGTT		
PMQRs			
*qnrA*	F: ATTTCTCACGCCAGGATTTG	516	Colobatiu et al.,2015 [19]
	R: GATCGGCAAAGGTTAGGTCA		
*qnrB*	F: GATCGTGAAAGCCAGAAAGG	476	Colobatiu et al.,2015 [19]
	R: ACGATGCCTGGTAGTTGTCC		
*qnrD*	F: CGAGATCAATTTACGGGGAATA	656	Cui et al.,2015 [13]
	R: AACAAGCTGAAGCGCCTG		
*qnrS*	F: ACGACATTCGTCAACTGCAA	417	Colobatiu et al.,2015 [19]
	R: TAAATTGGCACCCTGTAGGC		
*aac(6′)-Ib-cr*	F: CCCGCTTTCTCGTAGCA	544	Colobatiu et al.,2015 [19]
	R: TTAGGCATCACTGCGTCTTC		
*qepA*	F: CGTGTTGCTGGAGTTCTTC	403	Colobatiu et al.,2015 [19]
	R: CTGCAGGTACTGCGTCATG		
QRDR			
*gyrA*	F: TACACCGGTCAACATTGAGG	648	Hu et al.,2007 [20]
	R: TTAATGATTGCCGCCGTCGG		
*gyrB*	F: TGAAATGACCCGCCGTAAAGG	309	Hu et al.,2007 [20]
	R: GCTGTGATAACGCAGTTTGTCCGGG		
*parC*	F: GTACGTGATCATGGACCGTG	531	Hu et al.,2007 [20]
	R: TTCGGCTGGTCGATTAATGC		
*parE*	F: ATGCGTGCGGCTAAAAAAGTG	290	Hu et al.,2007 [20]
	R: TCGTCGCTGTCAGGATCGATAC		

### PFGE

Genotypes and transmission patterns were determined by performing PFGE according to the method described in a previous study [19]. *S. flexneri* isolates were digested with the restriction enzyme *Not*I (TaKaRa, Japan) at 37 °C for 3 h to generate a DNA fingerprinting profile. *Salmonella enterica* serotype Braenderup strain H9812 was digested with *Xba*I (TaKaRa, Japan) and used as a molecular size standard. Electrophoresis was performed on the CHEF Mapper XA system (Bio-Rad) with a 1% agarose SeaKem Gold gel (Lonza, USA). Electrophoretic parameters were determined by performing multiple screening runs and included switching times of 2.16 to 54.17 s, a voltage of 6 v/cm, a 120° angle and a run time of 21 h. PFGE images were obtained using a Universal Hood II (Bio-RAD, USA) and analyzed using BioNumerics software version 7.1 (Applied Maths, Sint-Martens-Latem, Belgium). A clustering tree that indicated relative genetic similarity was constructed using UPGMA (Unweighted Pair Group Method with Arithmetic Mean) and the Dice-predicted similarity value with a 1.0% pattern optimization and 1.5% band position tolerance.

## Results

### Bacterial isolation and identification

**Fig. 1:**
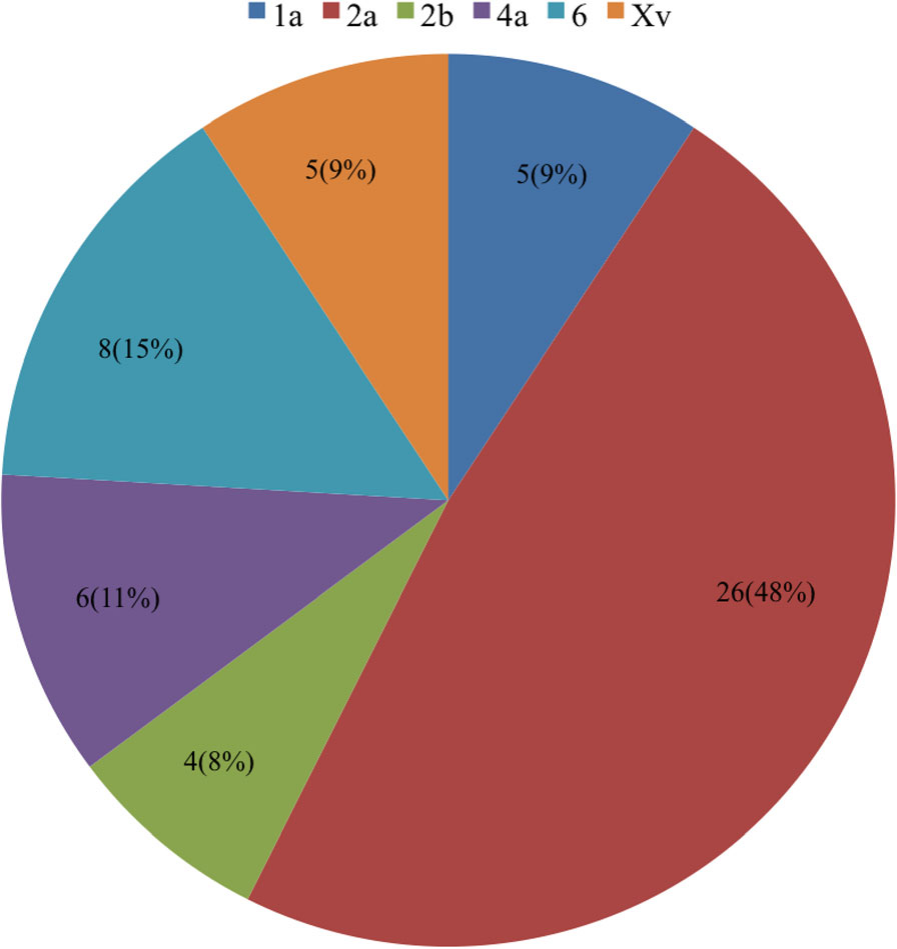
*S. flexneri* serotypes collected from 2014 to 2016

**Fig. 2:**
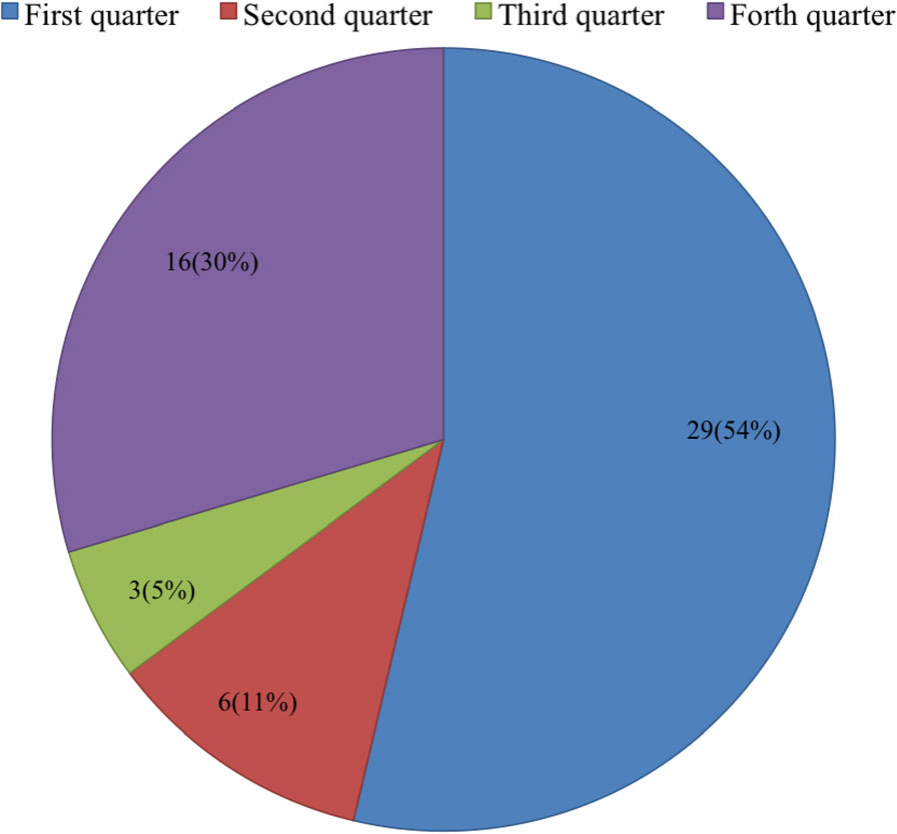
Number of *S. flexneri* isolated from different quarters

During our epidemiological survey of *Shigella*, we collected 873 fecal samples from calves with diarrhea and obtained 54 *S. flexneri* isolates from five provinces in northwest China from 2014 to 2016. Isolate information is shown in detail in Table 2. Among the 54 *S. flexneri* isolates, there were six serotypes: five (9.26%) isolates were 1a, twenty-six (48.15%) isolates were 2a, four (7.41%) isolates were 2b, six (11.11%) isolates were 4a, eight (14.81%) isolates were 6, and five (9.26%) isolates were Xv (Fig. 1). Our surveillance of the Gansu isolates identified all of the serotypes, except 4a. All 4a serotypes were isolated from Shanxi, while all Xv and 1a serotypes were from Gansu. Additionally, serotype 2a was widely isolated from each province, with the exception of Xinjiang, and serotype 6 was found only in yaks. Interestingly, *Shigella* was primarily isolated in the first quarter and fourth quarter, accounting for 54% (29/54) and 30% (16/54), respectively (Fig. 2).

**Table 2:** Strain information of *S. flexneri* isolates from diarrheal calves, 2014 to 2016

Strain name	Serotype	Isolation year	Origin	Province
TYSF1412001	2a	2014	Yak	Tibet
GBSF1412056	2a	2014	Beef cattle	Gansu
GBSF1501026	2a	2015	Beef cattle	Gansu
GBSF1501071	Xv	2015	Beef cattle	Gansu
GYSF1501076	6	2015	Yak	Gansu
QYSF1501088	6	2015	Yak	Qinghai
XBSF1501093	2b	2015	Beef cattle	Xinjiang
GBSF1501105	2a	2015	Beef cattle	Gansu
SBSF1501123	4a	2015	Beef cattle	Shanxi
QYSF1502130	6	2015	Yak	Qinghai
GBSF1502176	2a	2015	Beef cattle	Gansu
GYSF1502197	6	2015	Yak	Gansu
SBSF1502219	4a	2015	Beef cattle	Shanxi
XBSF1502236	2b	2015	Beef cattle	Xinjiang
GBSF1503241	2a	2015	Beef cattle	Gansu
GYSF1503270	1a	2015	Yak	Gansu
GBSF1503288	1a	2015	Beef cattle	Gansu
GBSF1505314	2a	2015	Beef cattle	Gansu
SBSF1505331	2a	2015	Beef cattle	Shanxi
GBSF1506340	Xv	2015	Beef cattle	Gansu
GBSF1507358	1a	2015	Beef cattle	Gansu
GBSF1509369	2a	2015	Beef cattle	Gansu
GBSF1510375	2a	2015	Beef cattle	Gansu
GBSF1510390	2a	2015	Beef cattle	Gansu
QYSF1511395	2a	2015	Yak	Qinghai
GBSF1511401	2a	2015	Beef cattle	Gansu
GYSF1511409	2a	2015	Yak	Gansu
SBSF1512413	4a	2015	Beef cattle	Shanxi
GBSF1512419	2b	2015	Beef cattle	Gansu
GBSF1512425	2a	2015	Beef cattle	Gansu
GBSF1512433	2a	2015	Beef cattle	Gansu
GBSF1601015	Xv	2016	Beef cattle	Gansu
GBSF1601024	Xv	2016	Beef cattle	Gansu
TYSF1601031	2b	2016	Yak	Tibet
GBSF1601064	2a	2016	Beef cattle	Gansu
GYSF1601073	6	2016	Yak	Gansu
GBSF1602082	2a	2016	Beef cattle	Gansu
QYSF1602094	6	2016	Yak	Qinghai
GBSF1602098	2a	2016	Beef cattle	Gansu
GBSF1602103	2a	2016	Beef cattle	Gansu
SBSF1603115	4a	2016	Beef cattle	Shanxi
SBSF1603121	4a	2016	Beef cattle	Shanxi
GBSF1603138	2a	2016	Beef cattle	Gansu
GBSF1603149	2a	2016	Beef cattle	Gansu
QYSF1603158	6	2016	Yak	Qinghai
SBSF1604173	2a	2016	Beef cattle	Shanxi
SBSF1604195	4a	2016	Beef cattle	Shanxi
GBSF1605203	Xv	2016	Beef cattle	Gansu
GBSF1608241	2a	2016	Beef cattle	Gansu
GYSF1610256	2a	2016	Yak	Gansu
GYSF1610266	6	2016	Yak	Gansu
GBSF1610275	1a	2016	Beef cattle	Gansu
GBSF1611283	1a	2016	Beef cattle	Gansu
GBSF1611290	2a	2016	Beef cattle	Gansu

### Antimicrobial susceptibility testing

**Fig. 3:**
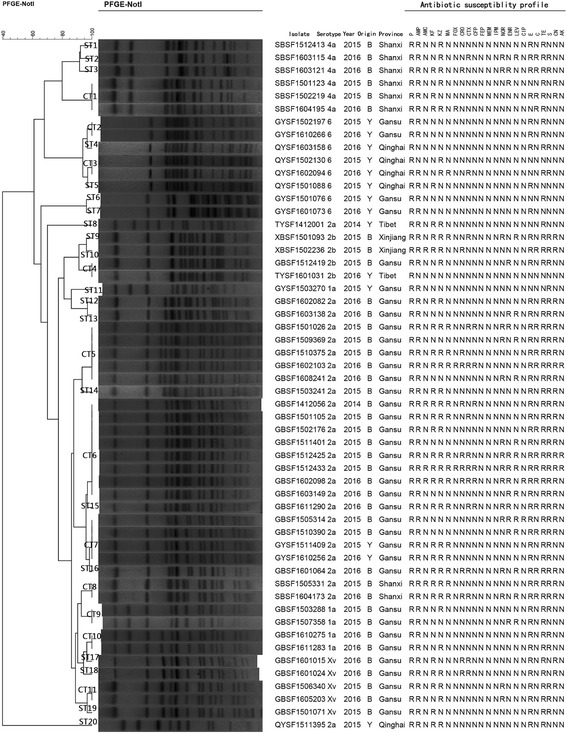
PFGE dendrogram and antibiotic susceptibility profile of 54 *Not*I-digested *S. flexneri*. B = Beef cattle; Y = Yak. R = Resistance; N = Sensitive and Intermediary

The 54 *S. flexneri* isolates were examined for susceptibility to 23 antibiotics. More than 50% of isolates were resistant to 8 antibiotics. Among them, resistance to P (54/54, 100%), AMP (51/54, 94.44%) and TE (49/54, 90.74) was most common, followed by E (46/54, 85.19%), S (38/54, 70.37%), KZ (34/54, 62.96%), KF (29/54, 53.70%) and CN (29/54, 53.70%). None of the isolates were resistant to IMP, MEM and the fourth-generation cephalosporin FEP. In addition, although a certain number of isolates were resistant to second- and third-generation cephalosporins (MA, FOX, CRO, CTX and CFP) and fluoroquinolones (CIP, NOR, ENR and LEV), these comprised no more than 30% of the total number of isolates, and the resistance rate was lower than those of other antibiotics (Table 3, Fig. 3).

**Table 3:** Statistical analysis of the results of antimicrobial susceptibility to 23 antibiotics for 54 *S. flexneri*

Antibiotic	Antimicrobial resistance rate No. (%)					
	Total (*n* = 54)	Gansu (*n* = 37)	Shanxi (*n* = 8)	Xinjiang (*n* = 2)	Qinghai (*n* = 5)	Tibet (*n* = 2)
Penicillin G (P)	54 (100%)	37 (100%)	8 (100%)	2 (100%)	5 (100%)	2 (100%)
Ampicillin (AMP)	51 (94.44%)	37 (100%)	8 (100%)	2 (100%)	3 (60%)	1 (50%)
Amoxycillin/Clavulanic acid (AMC)	5 (9.62%)	3 (8.11%)	1 (12.5%)	1 (50%)	0	0
Cephalothin (KF)	29 (53.70%)	19 (51.35%)	5 (62.5%)	2 (100%)	2 (40%)	1 (50%)
Cephazolin (KZ)	34 (62.96%)	21 (56.76%)	7 (87.5%)	2 (100%)	3 (60%)	1 (50%)
Cefamandole (MA)	16 (29.63%)	12 (32.43%)	2 (25%)	1 (50%)	1 (20%)	0
Cefoxitin (FOX)	3 (5.56%)	2 (5.41%)	1 (12.5%)	0	0	0
Ceftriaxone (CRO)	12 (22.22%)	9 (24.32%)	2 (25%)	1 (50%)	0	0
Cefotaxime (CTX)	14 (25.93%)	10 (27.03%)	2 (25%)	1 (50%)	1 (20%)	0
Cefoperazone (CFP)	6 (11.11%)	6 (16.22%)	0	0	0	0
Cefepime (FEP)	0	0	0	0	0	0
Meropenem (MEM)	0	0	0	0	0	0
Imipenem (IPM)	0	0	0	0	0	0
Norfloxacin (NOR)	16 (29.63%)	12 (32.43%)	3 (37.5%)	1 (50%)	0	0
Enrofloxacin (ENR)	13(24.07%)	11 (29.73%)	2 (25%)	0	0	0
Levofloxacin (LEV)	14 (25.93%)	11 (29.73%)	2 (25%)	1 (50%)	0	0
Ciprofloxacin (CIP)	2 (3.70%)	2 (5.41%)	0	0	0	0
Erythromycin (E)	46 (85.19%)	35 (94.59%)	6 (75%)	2 (100%)	3 (60%)	0
Tetracycline (TE)	49 (90.74%)	35 (94.59%)	8 (100%)	2 (100%)	3 (60%)	1 (50%)
Chloramphenicol (C)	17 (31.48%)	10 (27.03%)	6 (75%)	1 (50%)	0	0
Streptomycin (S)	38 (70.37%)	30 (81.08%)	4 (50%)	2 (100%)	2 (40%)	0
Gentamicin (CN)	29 (53.70%)	23 (62.16%)	4 (50%)	2 (100%)	0	0
Amikacin (AK)	3 (5.56%)	3 (8.11%)	0	0	0	0

Remarkably, all 26 *S. flexneri* 2a isolates demonstrated varying degrees of resistance to cephalosporins and/or fluoroquinolones and exhibited multidrug resistance (MDR). The *S. flexneri* 2a isolates were resistant to 14 diverse cephalosporins/fluoroquinolones. Among them, 73.06% (19/26) of isolates were resistant to cephalosporin, 53.85% (14/26) of isolates were resistant to fluoroquinolones, and 26.92% (7/26) of isolates were resistant to both cephalosporin and fluoroquinolones. Furthermore, isolate GBSF1512433 was resistant to all cephalosporins (with the exception of FEP) and fluoroquinolones (with the exception of CIP). Compared with the *S. flexneri* 2a isolates collected from beef calves, the 4 yak isolates were sensitive to most cephalosporins and fluoroquinolones but resistant to KF, KZ and MA (Table 4).

**Table 4:** Statistical analysis of the cephalosporin and/or fluoroquinolone susceptibility for 26 *S. flexneri* 2a

Cephalosporin and/or Fluorquinolones resistance spectrum	Cephalosporin and/or Fluorquinolones resistance rate No. (%)				
	Total (*n* = 26)	Gansu (*n* = 22^a^)	Shanxi (*n* = 2)	Qinghai (*n* = 1^a^)	Tibet (*n* = 1^a^)
KF/KZ	5 (19.23%)	3 (13.64%)	0	1^a^ (100%)	1^a^ (100%)
KF/KZ/MA	3 (11.54%)	2^a^ (9.09%)	1 (50%)	0	0
KF/KZ/MA/CRO	1 (3.85%)	1 (4.55%)	0	0	0
KF/KZ/MA/CTX	1 (3.85%)	1 (4.55%)	0	0	0
KF/KZ/MA/CRO/CFP	1 (3.85%)	1 (4.55%)	0	0	0
KF/KZ/MA/FOX/CRO/CTX/CFP	1 (3.85%)	1 (4.55%)	0	0	0
NOR/LEV	3 (11.54%)	3 (13.64%)	0	0	0
ENR/LEV	3 (11.54%)	3 (13.64%)	0	0	0
NOR/ENR/LEV/CIP	1 (3.85%)	1 (4.55%)	0	0	0
KF/KZ/MA/CRO/CTX/NOR/LEV	2 (7.69%)	1 (4.55%)	1 (50%)	0	0
KF/KZ/MA/CRO/CTX/NOR/ENR/LEV	1 (3.85%)	1 (4.55%)	0	0	0
KF/KZ/MA/CTX/CFP/CIP/NOR/ENR	2 (7.69%)	2 (9.09%)	0	0	0
KF/KZ/MA/CRO/CTX/CFP/NOR/ENR/CIP	1 (3.85%)	1 (4.55%)	0	0	0
KF/KZ/MA/FOX/CRO/CTX/CFP/NOR/ENR/LEV	1 (3.85%)	1 (4.55%)	0	0	0

^a^a yak origin *S. flexneri* 2a isolate

### ARGs analysis of cephalosporin- and/or fluoroquinolone-resistant *S. flexneri* 2a isolates

In this study, only three β-lactamase gene types (*bla*_*OXA*-*1*_, *bla*_*TEM*-*1*_ and *bla*_*CTX*-*M*-*14*_) were identified among the 19 cephalosporin-resistant *S. flexneri* 2a isolates (Table 5). All isolates harbored *bla*_*TEM*-*1*_ type ARGs (100%), 15 isolates harbored *bla*_*OXA*-*1*_ (15/19, 78.95%), and 14 harbored *bla*_*CTX*-*M*-*14*_ (14/19, 73.68%). In total, 63.16% (12/19) of isolates harbored three β-lactamase gene types. All *S. flexneri* 2a isolates from yaks were negative for *bla*_*CTX*-*M*_ type ARGs.

**Table 5:** Antimicrobial spectrum and ARGs analysis of *S. flexneri* 2a with resistance to cephalosporin

Strain name	Antimicrobial spectrum	ARGs in plasmid		
		*TEM*	*OXA*	*CTX*-*M*-*9*
TYSF1412001	KF/KZ	*TEM*-*1*	*OXA*-*1*	----
QYSF1511395	KF/KZ	*TEM*-*1*	----	----
GBSF1503241	KF/KZ	*TEM*-*1*	*OXA*-*1*	*CTX*-*M*-*14*
GBSF1502176	KF/KZ	*TEM*-*1*	*OXA*-*1*	*CTX*-*M*-*14*
GBSF1602082	KF/KZ	*TEM*-*1*	----	*CTX*-*M*-*14*
SBSF1505331	KF/KZ/MA	*TEM*-*1*	*OXA*-*1*	*CTX*-*M*-*14*
GYSF1511409	KF/KZ/MA	*TEM*-*1*	----	----
GYSF1610256	KF/KZ/MA	*TEM*-*1*	*OXA*-*1*	----
GBSF1510375	KF/KZ/MA/CRO	*TEM*-*1*	*OXA*-*1*	*CTX*-*M*-*14*
GBSF1501105	KF/KZ/MA/CTX	*TEM*-*1*	*OXA*-*1*	*CTX*-*M*-*14*
GBSF1412056	KF/KZ/MA/CRO/CFP	*TEM*-*1*	*OXA*-*1*	*CTX*-*M*-*14*
GBSF1602103	KF/KZ/MA/FOX/CRO/CTX/CFP	*TEM*-*1*	*OXA*-*1*	*CTX*-*M*-*14*
GBSF1601064	KF/KZ/MA/CRO/CTX/NOR/LEV	*TEM*-*1*	----	*CTX*-*M*-*14*
SBSF1604173	KF/KZ/MA/CRO/CTX/NOR/LEV	*TEM*-*1*	*OXA*-*1*	*CTX*-*M*-*14*
GBSF1611290	KF/KZ/MA/CTX/CFP/NOR/ENR	*TEM*-*1*	*OXA*-*1*	*CTX*-*M*-*14*
GBSF1501026	KF/KZ/MA/CTX/CFP/NOR/ENR	*TEM*-*1*	*OXA*-*1*	*CTX*-*M*-*14*
GBSF1512425	KF/KZ/MA/CRO/CTX/NOR/ENR/LEV	*TEM*-*1*	*OXA*-*1*	----
GBSF1602098	KF/KZ/MA/CRO/CTX/CFP/NOR/ENR/CIP	*TEM*-*1*	*OXA*-*1*	*CTX*-*M*-*14*
GBSF1512433	KF/KZ/MA/FOX/CRO/CTX/CFP/NOR/ENR/LEV	*TEM*-*1*	*OXA*-*1*	*CTX*-*M*-*14*

Both PMQR genes and SNPs in QRDRs were identified for 14 quinolone-resistant isolates (Table 6). According to the PCR results, all quinolone-resistant isolates were positive for *aac(6*′*)-Ib-cr* but negative for *qepA*, except strain GBSF1602098. Only five (5/14, 35.71%) strains isolated from Gansu harbored *qnrS*, and no isolate harbored all three ARGs simultaneously. The point mutations in the QRDR genes play important roles in determining quinolone and/or fluoroquinolone resistance [24]. In the present study, we successfully amplified all four QRDR genes and compared them to reference sequences. We found two point mutations in *gyrA* and one point mutation each in *gyrA* and *parC* (Table 6). All quinolone-resistant strains carried mutations that altered the amino acid sequences of *gyrA* (S83 L) and *parC* (S80I). In addition, each strain carried the mutation 87 (D → N or Y) in *gyrA*, with the exception of GBSF1510390. Interestingly, GBSF1505314 and GBSF1602098 harbored the *gyrA* D87Y mutation, which confers resistance to ciprofloxacin.

**Table 6:** Antimicrobial spectrum and amino acid types in QRDR and PMQRs genes analysis of *S. flexneri* 2a with resistance to fluoroquinolones

Strain name	Antimicrobial spectrum	QRDR			ARGs in plasmid		
		*gyrA*		*parC*	*aac(6*′*)-Ib-cr*	*qnrS*	*qepA*
		83	87	80			
GBSF1509369	NOR/LEV	S83 L	D87N	S80I	+	−	−
GBSF1511401	NOR/LEV	S83 L	D87N	S80I	+	−	−
GBSF1608241	NOR/LEV	S83 L	D87N	S80I	+	−	−
GBSF1510390	ENR/LEV	S83 L	D87D	S80I	+	+	−
GBSF1603138	ENR/LEV	S83 L	D87N	S80I	+	−	−
GBSF1603149	ENR/LEV	S83 L	D87N	S80I	+	−	−
GBSF1505314	NOR/ENR/LEV/CIP	S83 L	D87Y	S80I	+	+	−
GBSF1601064	KF/KZ/MA/CRO/CTX/NOR/LEV	S83 L	D87N	S80I	+	−	−
SBSF1604173	KF/KZ/MA/CRO/CTX/NOR/LEV	S83 L	D87N	S80I	+	−	−
GBSF1611290	KF/KZ/MA/CTX/CFP/NOR/ENR	S83 L	D87N	S80I	+	−	−
GBSF1501026	KF/KZ/MA/CTX/CFP/NOR/ENR	S83 L	D87N	S80I	+	+	−
GBSF1512425	KF/KZ/MA/CRO/CTX/NOR/ENR/LEV	S83 L	D87N	S80I	+	+	−
GBSF1602098	KF/KZ/MA/CRO/CTX/CFP/NOR/ENR/CIP	S83 L	D87Y	S80I	+	−	+
GBSF1512433	KF/KZ/MA/FOX/CRO/CTX/CFP/NOR/ENR/LEV	S83 L	D87N	S80I	+	+	−

+: Presence corresponding genes

-: Absence corresponding genes

### PFGE pattern analysis

PFGE was performed to determine the genetic relatedness among the isolates and to study the molecular epidemiology in specific geographical regions [25]. The PFGE patterns of the 54 *Not*I-digested *S. flexneri* isolates were heterogeneous, and multiple PFGE patterns were present among the strains. Thus, diverse factors such as geography and environment may affect PFGE patterns. At an 80% similarity level, *S. flexneri* isolates generated 31 reproducible and unique PFGE patterns, including 11 common types (CT) and 20 single types (ST) (Fig. 3).

Among all isolates, the majority of *S. flexneri* 2a (26/54, 48.15%) isolates were classified into 11 PFGE patterns (4 CT and 7 ST). These PFGE patterns were closely related to each other, except the Tibet (TYSF1412001) and Qinghai (QYSF1511395) isolates, suggesting the strains isolated from different geographical locations exhibit diverse PFGE patterns and a capricious genetic diversity.

## Discussion

ARGs are widespread and cause problems when present in pathogens [26]. Over the past decade, MDR *Shigella* has been reported in many countries [27]. However, only a few studies have described the prevalence of *Shigella* in animals worldwide. In the present study, we investigated the epidemiology of *S. flexneri* in cows in northwest China. During a 2-year survey, 54 *S. flexneri* isolates were obtained. Unfortunately, 16S rRNA gene sequence analysis does not effectively distinguish between closely related strains in a superfamily, such as *Shigella* and *E. coli* [28], and conventional biochemical and serological techniques are also insufficient. Therefore, PFGE was utilized to analyze the molecular characteristics of these isolates, to determine the relatedness among isolates and to study the molecular epidemiology in specific geographical regions. The clustering results allowed us to analyze the epidemiological trends of *S. flexneri*. Characterization of these isolates will be helpful for clinical diagnosis, treatment, prevention and the control of shigellosis [15].

Antimicrobial resistance has emerged as a serious problem [29], particularly for conventional, older-generation antibiotics such as P, AMP, TE, and E. According to the results of our antimicrobial susceptibility tests, cephalosporin and fluoroquinolone resistance rates in our isolates were higher than those in human isolates [19, 26]. Notably, the predominant *S. flexneri* 2a isolates were all resistant to cephalosporins, fluoroquinolones and multiple antibiotics. Two isolates (GBSF1505314 and GBSF1602098) were also resistant to ciprofloxacin, which is the first-line antibiotic treatment for shigellosis. The universal emergence of resistant and MDR strains in animals may be attributable to the unrestricted and excessive use of antibiotics in veterinary clinics. The widespread presence of MDR strains has reduced the selectivity of clinical medications to treat shigellosis [30]. Notably, our PFGE dendrogram showed various genetic patterns for *S. flexneri*, and there were diverse resistance profiles associated with each pattern. Based on these results, *S. flexneri* has the ability to adapt to the selective pressures of different antibiotics.

The high levels of resistance of S*. flexneri* 2a to cephalosporin/fluorquinolones, which are the most effective treatments for severe gastrointestinal infections caused by pathogenic bacteria, prompted us to study potential molecular resistance mechanisms. The emergence of ESBL-producing *Shigella* spp. has been observed in many countries [31]. In the current study, only 3 ARG genotypes (*bla*_*OXA*-*1*_, *bla*_*TEM*-*1*_ and *bla*_*CTX*-*M*-*14*_) were detected. Among them, the *bla*_*TEM*-*1*_ gene was detected in all 19 cephalosporin-resistant isolates. In total, 174 *bla*_*TEM*_ variants resistant to penicillin and other ß-lactamase antibiotics have been recorded. *TEM*-*1* confers resistance to ampicillin and cephalothin [32]. *bla*_*OXA*_-type ARGs are class D β-lactamases, which were named for their ability to hydrolyze oxacillin [32]. Initially, *bla*_*OXA*_-beta-lactamases were reported in *P. aeruginosa*, although now the *bla*_*OXA*_ gene has been detected in plasmids and integrons in many Gram-negative organisms [32, 33]. According to some studies, the probable host preference for *bla*_*OXA*_-type β-lactamase is *S. flexneri* [34]. In the present study, 15/19 (78.95%) isolates harbored *bla*_*OXA*_- type genes, and sequencing results indicated that all the *bla*_*OXA*_ genes were *bla*_*OXA*-*1*_. Additionally, *bla*_*CTX*-*M*_ has become one of the most prevalent extended-spectrum-β-lactamases (ESBLs) [35]. This gene was widely harbored by S*. flexneri* 2a isolated from beef cattle. Interestingly, all *S. flexneri* 2a isolated from yaks were negative for *bla*_*CTX*-*M*_ type ARGs.

Fluoroquinolones are highly effective for the treatment of shigellosis worldwide [36]. The primary mechanism of quinolone resistance involves the accumulation of sequential mutations in QRDRs that encode DNA gyrase and topoisomerase IV [37]. The most prevalent mutations in *Shigella* spp. are the point mutations in *gyrA* codons 83, 87 and 211, and *parC* codon 80 [38, 39]. Novel mutations in QRDRs are also being discovered [39]. In the present study, three mutations in *gyrA* codon 83 (S → L) and/or 87 (D → N or Y) and *parC* codon 80 (S → I) were detected in each fluoroquinolone-resistant isolate. All substitutions are responsible for reduced affinity. In addition, the amino acid diversity at the same position may lead to different levels of quinolone resistance [40, 41]. *GyrA* D87Y mutations were detected in only two ciprofloxacin-resistant isolates. However, the role of this mutation in ciprofloxacin resistance is unclear and requires further investigation.

Over the past few years, PMQR determinants have been deemed the most common ARGs in Enterobacteriaceae worldwide [42]. PMQR determinants mediate only low-level quinolone resistance. However, these resistance genes are usually associated with mobile or transposable elements that allow for dissemination among Enterobacteriaceae. In addition, the presence of PMQR genes may facilitate the selection of QRDR mutations that result in higher levels of quinolone resistance [37, 43, 44]. The *aac(6*′*)-Ib-cr* gene encodes an acetyltransferase that is known to reduce quinolone activity. In the present study, all 14 isolates resistant to fluoroquinolones were positive for *aac(6*′*)-Ib-cr*, indicating the *aac(6*′*)-Ib-cr* gene is widespread in *S. flexneri* 2a. Compared with the *aac(6*′*)-Ib-cr* gene, the transmembrane segment efflux pump *qepA* gene was scarcely detected in *Shigella,* and we found only one ciprofloxacin-resistant isolate that was *qepA*-positive. The qnr family (which includes the first PMQR genes) contains a variety of subtypes, including *qnrA*, *qnrB*, *qnrC*, *qnrD* and *qnrS* and several *qnr* family genes that have been reported in *Shigella* [39, 45]. The *Qnr* proteins protect DNA gyrase against quinolones and facilitate the selection of QRDR mutations that improve resistance to these antimicrobials.

## Conclusion

In conclusion, cephalosporin and/or fluoroquinolone resistance in *Shigella* has been widely reported. To increase our understanding of *Shigella* in cattle, we investigated *Shigella* in calves with diarrhea and analyzed the genetic relatedness, antimicrobial susceptibility, QRDR mutations, and prevalence of PMQR and ß-lactamase in *S. flexneri* 2a isolates from five provinces in northwest China. However, this study also had limitations, including the lack of a systematic surveillance system to prospectively or retrospectively detect and analyze shigellosis in veterinary clinics. Furthermore, we are unable to effectively monitor and control antibiotic abuse and the resulting spread of ARGs. Therefore, it is essential to continually monitor rates of shigellosis and the development of resistance patterns.
